# Green and red fluorescent strains of *Xenorhabdus griffiniae* HGB2511, the bacterial symbiont of the nematode *Steinernema hermaphroditum* (India)

**DOI:** 10.17912/micropub.biology.001064

**Published:** 2024-02-03

**Authors:** Nadia M. St. Thomas, Tyler G. Myers, Omar S. Alani, Heidi Goodrich-Blair, Jennifer K. Heppert

**Affiliations:** 1 Maryville College, Maryville, Tennessee, United States; 2 Microbiology, University of Tennessee at Knoxville, Knoxville, Tennessee, United States

## Abstract

*Steinernema* entomopathogenic nematodes form specific, obligate symbiotic associations with gram-negative, gammaproteobacteria members of the *Xenorhabdus* genus. Together, the nematodes and symbiotic bacteria infect and kill insects, utilize the nutrient-rich cadaver for reproduction, and then reassociate, the bacteria colonizing the nematodes’ anterior intestines before the nematodes leave the cadaver to search for new prey. In addition to their use in biocontrol of insect pests, these nematode-bacteria pairs are highly tractable experimental laboratory models for animal-microbe symbiosis and parasitism research. One advantageous feature of entomopathogenic nematode model systems is that the nematodes are optically transparent, which facilitates direct observation of nematode-associated bacteria throughout the lifecycle. In this work, green- and red-fluorescently labeled *X. griffiniae* HGB2511 bacteria were created and associated with their *S* . *hermaphroditum* symbiotic nematode partners and observed using fluorescence microscopy. As expected, the fluorescent bacteria were visible as a colonizing cluster in the lumen of the anterior intestinal caecum of the infective stage of the nematode. These tools allow detailed observations of *X. griffiniae* localization and interactions with its nematode and insect host tissues throughout their lifecycles.

**Figure 1. GFP and TurboRFP were inserted into the att Tn 7 site in X. griffiniae HGB2511:**
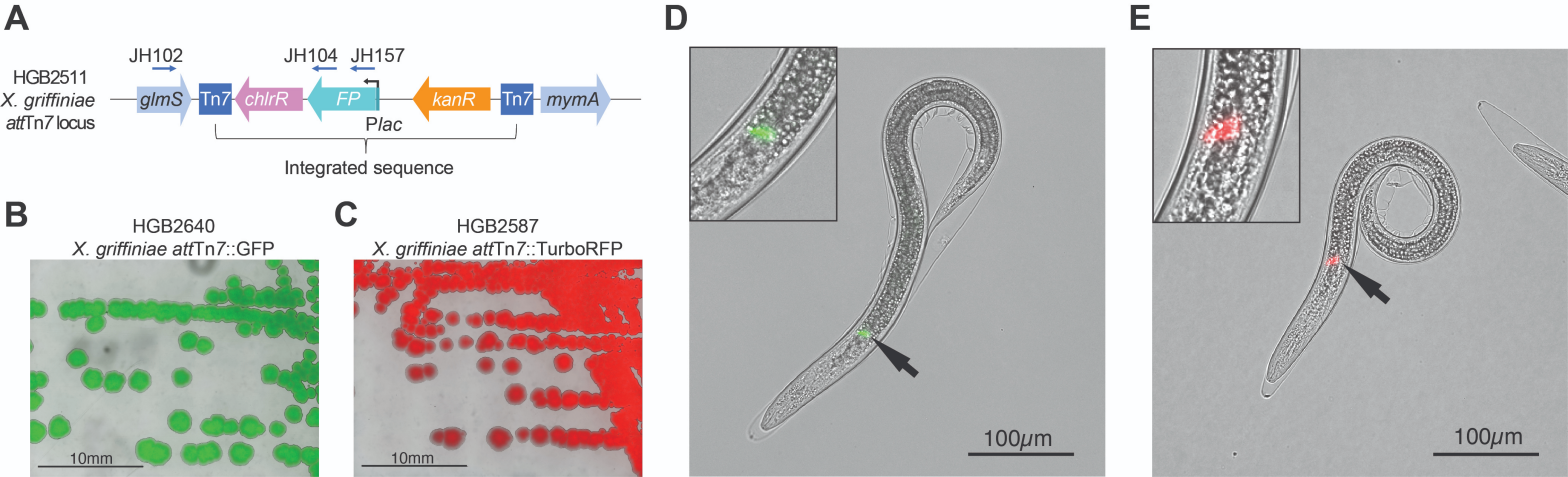
A) A schematic diagram of the *att* Tn *7* ­­­­locus and integrated sequence including fluorescent protein transgene and selectable markers. Primers used to confirm the insertion are indicated _­­_ by arrows above the locus. Exconjugant *X. griffiniae* bacteria expressing GFP or TurboRFP were visualized by fluorescence microscopy during growth as colonies on agar medium (B and C) or colonizing the intestines of *S. hermaphroditum* infective juvenile (IJ) stage nematodes (merged images, D and E). Arrows indicate the site of colonization, the receptacle, a specialized structure at the anterior of the intestine. An enlarged view of the receptacle is also shown in the inset in the upper left of panels D and E.

## Description

*Steinernema* nematodes and *Xenorhabdus* bacteria form specific symbiotic partnerships. Although in natural ecosystems these symbioses are likely obligate, in a laboratory setting the nematodes and bacteria can be raised independently with economical bench-top husbandry (Cao et al. 2022) . This ability to separately grow host and symbiont allows for genetic modification of the bacterial symbiont and reintroduction of the modified strain to its nematode host (McMullen and Stock 2014) . Fluorescent labeling of other entomopathogenic nematode associated bacterial symbionts using genetically encoded fluorescent proteins has led to detailed characterizations of colonization, persistence/accommodation, and predation, as well as the transitions between these life cycle phases (Ciche and Goffredi 2007; Somvanshi et al. 2012; Martens, Heungens, and Goodrich-Blair 2003; Sugar et al. 2012; Chaston et al. 2013) . *Steinernema hermaphroditum* nematodes and *Xenorhabdus griffiniae* HGB2511 bacteria are an emerging animal-microbe model pair due to recent progress in cryopreservation of the nematodes, and development of genomic and genetic tools for both organisms (Cao et al. 2022; Schwartz et al. 2023; Cao 2023; Alani et al. 2023) .

To facilitate fluorescent labeling of *X. griffiniae* , we first created matched mini-Tn *7* donor plasmids carrying green and red fluorescent proteins. We introduced each donor plasmid construct and a helper plasmid carrying the Tn *7* transposase into *X. griffiniae* HGB2511 by conjugation, targeting insertion into the chromosome at *att* Tn *7* site of *X. griffiniae* HGB2511 ( Fig. 1A ) (Choi et al. 2005; Choi and Schweizer 2006; Alani et al. 2023) . The conserved *att* Tn *7* is located in an intergenic downstream of the *glmS* gene and is considered a neutral insertion site for the insertion of transgenes with chromosomal incorporation resulting in minimal impacts on organismal fitness (Peters and Craig 2001; Choi et al. 2005) . Candidate exconjugant colonies were initially screened for kanamycin resistance and visible colony fluorescence. After re-streaking candidates on selective media and isolating single colonies, constitutive expression of GFP and TurboRFP in *X. griffiniae* HGB2511 was confirmed using epifluorescence microscopy ( Figure 1B and C ). Insertion at the *att* Tn *7* site was confirmed by PCR, and the selected exconjugants were named HGB2640 ( *X. griffiniae att* Tn *7* ::GFP) and HGB2587 ( *X. griffiniae att* Tn *7* ::TurboRFP). To associate *S. hermaphroditum* nematodes with the fluorescent strains, axenic eggs were added to lawns of *X. griffiniae att* Tn *7* ::GFP and *X. griffiniae att* Tn *7* ::TurboRFP and grown for ~7 days at room temperature (Heungens, Cowles, and Goodrich-Blair 2002) . Both strains supported growth of the nematodes through at least two reproductive generations and emergence of the nematodes as infective juveniles (IJs), indicating that expression of GFP and TurboRFP did not grossly affect nematode reproduction and development. Epifluorescence microscopy revealed that, the IJ nematodes were colonized with green and red fluorescent bacteria in the expected location, the receptacle, a specialized structure in the anterior intestine ( Fig. 1D and E ) (Kim, Flores-Lara, and Stock 2012) .

*X. griffiniae* HGB2511 bacteria expressing GFP and TurboRFP from their respective integrated Tn *7* constructs supported nematode growth, colonized appropriate tissues, persisted within IJ nematodes, and allowed for infection and killing of insect larvae. A more detailed comparison will be required to understand if any subtle differences exist among the wild type and two fluorescent strains. The strains reported here will be useful tools to study the mechanisms and dynamics of the *S. hermaphroditum* and *X. griffiniae* HGB2511 symbiosis, including facilitating real-time *in vivo* and *in insecta* observation.

## Methods

***Strains and Primers***

Detailed descriptions of the strains and primer sequences used in this study can be found in Tables 1 and 2, respectively.

***Tn7 TurboRFP integration plasmid cloning***

To create GFP and TurboRFP expressing strains, we modified the mini-Tn *7* donor plasmid pURR25 (Ciche and Goffredi 2007) , replacing gfpmut3* (referred to as GFP) with TurboRFP (pturboRFP-B, Evrogen), creating the new donor plasmid pNST001 (Table 1). Briefly, the backbone of the pURR25 plasmid minus the GFP sequence was PCR amplified using primers JH163 and JH164, and Q5 polymerase (NEB), the product was digested with Dpn1 overnight at 37°C (NEB), and a 5-kb band was subsequently gel extracted and purified using the Zymoclean Gel DNA Recovery Kit (Zymo). The TurboRFP gene was amplified from HGB1625 (pturboRFP-B, Evrogen) using primers JH157 and JH158 (Table 2) and purified using the DNA Clean and Concentrator kit (Zymo). The PCR products were then assembled using HiFi Assembly Master Mix (NEB). The plasmid was confirmed by MscI restriction digest and sequencing.

***Conjugation of Tn7 integration plasmids into X. griffiniae***

An established protocol was used to conjugate the GFP donor plasmid (pURR25), TurboRFP donor plasmid (pNST001), and the Tn *7* transposon helper plasmid into *X. griffiniae* HGB2511 (Alani et al. 2023) (See Table 1 for strains carrying plasmids). Briefly, 5 mL cultures of the *E. coli* donor and recipient strains were grown overnight in Luria-Bertani broth stored in the dark (dark LB) with 150 µg/mL ampicillin and 0.3 mM diaminopimelic acid (DAP) included for HGB2432, and 50 µg/mL kanamycin and 0.3 mM DAP for HGB1262 and HGB2580. The overnight cultures were then subcultured at a 1:10 ratio and grown to an OD _600_ of ~1.0. *X. griffiniae* HGB2511, the *E. coli* carrying the Tn *7* helper plasmid HGB2432, and each of the *E. coli* donor strains HGB1262 (GFP), HGB2580 (TurboRFP) were mixed in an Eppendorf tube at the volumes of 900 µL, 300 µL, and 300 µL, pelleted, and the pellet was resuspended and spot-plated on LB agar plates supplemented with 0.1% pyruvate (LBP) and 0.3 mM DAP. The conjugation was incubated overnight at room temperature and kept in the dark, and then plated on LBP plates with 50 µg/mL kanamycin and 15 µg/mL chloramphenicol for selection. Exconjugants from each conjugation were confirmed to have GFP or TurboRFP fluorescence and patch plated on LBP with 50 µg/mL kanamycin and 15 µg/mL chloramphenicol. Candidates were then restreaked on whole LBP plates with 50 µg/mL kanamycin and 15 µg/mL chloramphenicol, single colonies were selected and grown in liquid culture, some of which was used to make glycerol stocks and some for genomic DNA extraction using Purelink Genomic DNA Mini Kit (Invitrogen). PCR using the genomic DNA samples was used to confirm insertion of the donor constructs at the Tn *7* site of the exconjugants (HGB2640, *X. griffiniae att* Tn *7* ::GFP confirmed with primers JH102 and JH104, and HGB2587, *X. griffiniae att* Tn *7* ::TurboRFP was confirmed with primers JH102 and JH157) ( Fig. 1A ; Table 2).

***Colonization of the S. hermaphroditum nematode host with X. griffiniae attTn7::GFP and X. griffiniae att:Tn7 Turbo-RFP***

*S. hermaphroditum* nematodes were grown on Lipid Agar plates (McMullen and Stock 2014) , seeded with lawns of *X. griffiniae* HGB2511 bacteria, and incubated for 72 h at room temperature. Axenic eggs were extracted from gravid adult nematodes using a standard bleaching protocol (McMullen and Stock 2014) . HGB2640 *X. griffiniae att* Tn *7* ::GFP and HGB2587 *X. griffiniae att* Tn *7* ::TurboRFP were seeded as lawns on Lipid Agar plates. Axenic eggs were added to the lawns (1000 eggs/plate), and the nematodes were grown at room temperature for ~7 d until the bacterial lawns appeared to be fully consumed and IJ stage nematodes were observed. The plates were then White trapped in 10 mls of water and IJs were collected when they emerged into the water after 7-10 d (Heungens, Cowles, and Goodrich-Blair 2002) . Bacteria and nematodes were imaged using a Keyence epifluorescence microscope with 4X, 20X, and 100X objectives, and Brightfield, GFP, and TexasRed filter sets.

## Reagents

**Table:** 

Table 1. Bacterial Strains used and made in this study				
Strain name	Genotype	Plasmid	Antibiotic markers*	Reference
HGB2511	*X. griffiniae* isolate 9 from *S. hermaphroditum* (India)	none		Cao et al., 2022
HGB2640	*X. griffiniae* isolate 9 *att* Tn *7:* :GFP	none	Kan; Chlr	This publication
HGB2587	*X. griffiniae* isolate 9 *att* :Tn *7* ::TurboRFP	none	Kan; Chlr	This publication
HGB1625	*E. coli * Top10 pturboRFP-B	pturboRFP‑B	Amp	Evrogen
HGB2578	*E. coli* (S17 λpir) pNST001	pNST001	Strep, Kan; Chlr	This publication
HGB2580	*E. coli* (S17 λpir, DAP dependent) pNST001	pNST001	Strep, Kan, Chlr; requires DAP	This publication
HGB1262	*E. coli* (BW29427 DAP dependent) pURR25	pURR25	Strep, Kan, Chlr; requires DAP	Ciche et al., 2007
HGB2432	*E. coli* (DAP dependent) with Tn *7* helper	pUX-BF13	Amp; requires DAP	Bao et al., 1991
*Key for abbreviations: Kan=kanamycin, Chlr= chloramphenicol, Amp=ampicillin, Strep=streptomycin, DAP=diaminopimelic acid.				

**Table:** 

Table 2. Primers used in this study		
Primer Number	Primer name	Primer sequence (5’-3’)
JH102	universal_glmS_Fwd_2	CACACGTAGAAGAGTTGATC
JH104	pURR25_GFP_Rev	TGGAAGCGTTCAACTAGCAGAC
JH157	TurboRFPF	ATGAGCGAGCTGATCAAG
JH158	TurboRFPR	TCATCTGTGCCCCAGTTTG
JH163	puRR25_R_2	CATGCTTAATTTCTCCTC
JH164	puRR25_F_2	GCTTAATTAGCTGAGC
